# Effect of 8% arginine toothpaste on Streptococcus mutans in patients undergoing fixed orthodontic treatment: randomized controlled trial

**DOI:** 10.1590/2177-6709.27.3.e2220322.oar

**Published:** 2022-07-04

**Authors:** Iman RAZEGHIAN-JAHROMI, Neda BABANOURI, Zahra EBRAHIMI, Hooman Zarif NAJAFI, Maryam SARBAZ, Nima MONTAZERI-NAJAFABADY

**Affiliations:** 1 Shiraz University of Medical Sciences, Cardiovascular Research Center (Shiraz, Iran).; 2 Shiraz University of Medical Sciences, Orthodontic Research Center (Shiraz, Iran).; 3 Shiraz University of Medical Sciences, Endocrinology and Metabolism Research Center (Shiraz, Iran).

**Keywords:** Arginine, Orthodontics, Prebiotics, L-arginine, Streptococcus mutans

## Abstract

**Objective:**

To assess the effect of toothpaste containing 8% arginine on *Streptococcus mutans* (*S. mutans*) in dental plaque around orthodontic brackets, and to draw a comparison with a regular fluoride toothpaste.

**Trial design:**

A single-center, parallel-arm, triple-blind, randomized controlled trial was conducted.

**Methods:**

The clinical trial was conducted at the Orthodontic Clinic, School of Dentistry, Shiraz University of Medical Sciences, Shiraz, Iran. Seventy-two patients (age range: 15-30 years) who required fixed orthodontic treatment were recruited and randomly assigned to arginine and fluoride groups. Randomization was performed using RANDOM.ORG online software, and the participants were divided into two parallel groups, with a 1:1 allocation ratio. Patients were requested to brush their teeth twice daily for 30 days with an experimental toothpaste. Plaque sampling was performed at two intervals, namely at the beginning of the study (T_0_) and 30 days later (T_1_). Real-time PCR was used to assess plaque samples in terms of the number of *S. mutans* surrounding stainless steel brackets in orthodontic patients. A triple-blind design was employed.

**Results:**

The baseline characteristics (age, sex, and the relative number of *S. mutans*) between the groups were similar (*p*>0.05). Only the arginine group showed a significant decrease in the relative number of bacteria between T_0_ and T_1_ (*p*=0.02).

**Conclusion:**

Arginine is an important prebiotic agent in maintaining healthy oral biofilms, and prevent dental caries during fixed orthodontic treatments.

**Trial registration:**

The trial was registered at the Iranian Registry of Clinical Trials (IRCT20181121041713N2), https://en.irct.ir/user/trial/42409/view.

## RESUMO

**Objetivo:**

Avaliar o efeito de um dentifrício contendo arginina a 8% no *Streptococcus mutans* (*S. mutans*) da placa bacteriana ao redor de braquetes ortodônticos, e compará-lo a um dentifrício fluoretado convencional.

**Desenho do estudo:**

Foi conduzido um estudo unicêntrico, com braços paralelos, triplo-cego, controlado e randomizado.

**Métodos:**

O ensaio clínico foi conduzido na Clínica de Ortodontia da Faculdade de Odontologia da Universidade de Ciências Médicas de Shiraz, no Irã. Setenta e dois pacientes (com idades variando de 15 a 30 anos) que necessitavam de tratamento ortodôntico com aparelho fixo foram recrutados e alocados aleatoriamente nos grupos arginina ou flúor. A randomização foi feita usando o programa on-line RANDOM.ORG, e os participantes foram divididos em dois grupos paralelos, com proporção de alocação de 1:1. Solicitou-se aos pacientes que escovassem os dentes duas vezes ao dia com a pasta experimental, durante 30 dias. Amostras da placa bacteriana foram coletadas em dois intervalos: ao começo do estudo (T_0_) e após 30 dias (T_1_). Um PCR em tempo real foi usado para avaliar as amostras de placa, em termos de números de *S. mutans* ao redor dos braquetes de aço inoxidável nos pacientes ortodônticos. Um desenho de estudo triplo-cego foi usado.

**Resultados:**

As características iniciais (idade, sexo e quantidade relativa de *S. mutans*) foram semelhantes entre os grupos (*p*>0,05). Apenas o grupo arginina mostrou uma redução significativa na quantidade de bactérias entre T_0_ e T_1_ (*p*=0,02).

**Conclusão:**

A arginina é um agente prebiótico importante na manutenção de biofilmes bucais saudáveis, e previne as cáries dentárias durante o tratamento ortodôntico com aparelho fixo.

**Registro do ensaio:**

O ensaio foi registrado no *Iranian Registry of Clinical Trials* (IRCT20181121041713N2), https://en.irct.ir/user/trial/42409/view.

## INTRODUCTION

Despite improvements in the design of orthodontic appliances, concerns exist about the treatment protocol and patient care during orthodontic treatment and the risk of enamel demineralization and white spot lesions. In terms of enamel demineralization during orthodontic treatment, incidences of up to 50% of patients have been reported.^1^ ^,^ ^2^ A variety of techniques have been proposed to prevent or reduce the occurrence of enamel demineralization during fixed orthodontic treatment, such as educating patients about dental plaque management, recommending the use of fluoride mouth rinse and dentifrice, prescribing probiotics and antibiotics, using fluoride-releasing restorative materials, and the use of nanoparticles, due to antibacterial properties.^1^ ^,^ ^3^ ^-^ ^6^

In recent years, biological methods such as antibiotics, antimicrobial therapy (with chlorhexidine, povidone-iodine, fluoride, and penicillin), and the use of live microbial dietary supplements or toothpastes have gained importance.^1^ ^,^ ^7^ ^,^ ^8^ Oral biofilms are highly organized communities of a physiologically and genetically diverse group of microorganisms, many of which are either commensal (i.e., overtly beneficial) or opportunistically pathogenic.^9^ It is well-known that the consumption of fermentable carbohydrates initiates caries formation by acidogenic bacteria living in the biofilm on tooth surface. A healthy biofilm can become cariogenic through a protracted presence of acid in the biofilm, which in turn promotes the growth of acid-tolerant bacteria (e.g., *Streptococcus mutans* and lactobacilli), leading to dental caries.^10^ ^,^ ^11^ In contrast, the presence of bacteria that are less acid-tolerant (e.g., *Streptococcus sanguinis* and *Streptococcus gordonii*) is associated with good oral health.^12^ ^,^ ^13^ Oral commensal bacteria stimulate an oral biofilm environment in various ways, to inhibit the presence of pathogenic species. Compared to *S. mutans,* commensal streptococci are less acid-tolerant and can catabolize urea using urease enzymes and/or arginine via the arginine deiminase system (ADS). As a result, ammonia, which alkalinizes the cytoplasm of the commensal, is released and thereby raises the pH in biofilm communities. Furthermore, hydrolysis of urea by ureases and arginine via ADS provides bioenergetic advantage to the commensal, which in turn improves the stability and health of biofilms.^9^

Prebiotics are compounds that have a positive effect on human health by selectively stimulating the growth or activity of beneficial microbes.^14^ Arginine is a prebiotic-based organic compound that, in combination with fluoride and calcium compounds, provides significant anti-caries benefits compared to matched formulations containing fluoride alone.^15^

Various studies have reported the beneficial effects of toothpaste containing arginine and fluoride on the de/remineralization balance. Compared to toothpaste with fluoride alone, this combination has superior efficacy in caries prevention.^16^ ^,^ ^17^ A previous *in vitro* study showed that the ecology of the biofilm bacteria grown under cariogenic conditions changes when exposed to L-arginine. In the presence of 1.5% exogenous L-arginine, the growth of *Streptococcus gordonii* (commensal) is more enhanced than of cariogenic *S. mutans*.^9^ Another study reported that toothpaste containing 1.5% arginine combined with 1,450 ppm fluoride provided greater levels of protection against the formation and progression of lesions, and promoted remineralization better than a dentifrice with 1,450 ppm fluoride alone.^10^

It has been shown that the level of acidogenic bacteria present in plaque, particularly *S. mutans,* is higher in orthodontic patients.^18^ ^,^ ^19^ However, to date, the possible effects of arginine toothpaste on dental biofilm in patients undergoing orthodontic treatment have not been investigated. Hence, the present randomized controlled trial was performed to evaluate possible additional benefits of arginine-containing toothpaste, compared with regular fluoride toothpaste.

### SPECIFIC OBJECTIVES OR HYPOTHESES

The primary purpose of the present study was to assess the effect of short-term usage of toothpaste containing 8.0% arginine on the level of *S. mutans* in plaque surrounding stainless steel brackets in orthodontic patients, and to draw a comparison with a toothpaste containing fluoride alone. The null hypothesis was that the 8.0% arginine toothpaste would not have any impact on the number of *S. mutans,* compared to the fluoride-containing toothpaste.

## MATERIAL AND METHODS

### TRIAL DESIGN

The present study was a single-center, triple-blind (patients, examiner, assessor) randomized controlled trial, with a 1:1 allocation ratio. The trial was conducted at the Orthodontic Clinic, School of Dentistry, Shiraz University of Medical Sciences, Shiraz, Iran. The study was approved by the Ethics Committee of Shiraz University of Medical Sciences (code: IR.SUMS.DENTAl.REC.1398.58) and registered at the Iranian Registry of Clinical Trials (IRCT20181121041713N2). The methods used remained unchanged throughout the study.

#### Participants, eligibility criteria, and settings

Participants were recruited among orthodontic patients attending the Orthodontic Clinic at the Dental Faculty of Shiraz University of Medical Sciences. The participants selection criteria are presented in Table 1. As part of the initial phase, the periodontal condition of all patients was assessed by the same periodontist. Prior to the study, the research goals, intervention methods, and probable risks and benefits were explained to the participants, the confidentiality of any disclosed information was guaranteed, and voluntary participation was emphasized. Written informed consent was obtained from all participants.

**Table 1:** inclusion and exclusion criteria.

Inclusion criteria	Exclusion criteria
Male and female patients	Diseases and medication use likely to affect dental biofilm
Age 15 to 30 years	Pregnancy
No significant medical history or drug use	Poor oral hygiene
No anti-inflammatory or antibiotic medications taken within 3 months prior to the study	Active periodontal disease
No chewing gum or mouthwash use in the week before and during the study	Severe crowding of anterior teeth or malposition of the lateral incisor likely to interfere with the cleaning of the tooth surface
No habit of brushing twice daily with fluoride toothpaste	Smoking
No history of periodontal therapy or current periodontal disease	History of mouth breathing
Probing depth less than 4 mm across the entire dentition	
No gingivitis or active carious lesion	
Gingival index and plaque index (Silness-Löe) value <1	

### SAMPLE SIZE CALCULATION

Assuming 50% reduction in *S. mutans* by fluoride-containing toothpaste,^1^ and additional 30% reduction by arginine-containing toothpaste, the required sample size (conventional alpha level of 0.05 and desired power [1-β] of 0.80) was 36 patients per group, yielding a total sample size estimate of 72 patients. 

### RANDOMIZATION (RANDOM NUMBER GENERATION, ALLOCATION CONCEALMENT, IMPLEMENTATION)

The block randomization method was used with a block size of 6 (RANDOM.ORG online software) to allocate patients to a control and an intervention group with a 1:1 ratio. Subsequently, the random sequences to either fluoride or arginine group were concealed in opaque, sealed envelopes and shuffled before the intervention, to increase the unpredictability of the random allocation sequence and avoid selection bias. Each patient was asked to pick an envelope for allocation to either the arginine or fluoride toothpaste group. 

### BLINDING

Identical tubes were used for both the arginine and fluoride toothpaste. The color-coding of the tubes was only known to the first author, and not to the others involved in the study (the clinicians, patients, laboratory technicians, and the statistician).

### INTERVENTION

*Sample preparation:* Fixed-appliance treatment of all patients was carried out by the same orthodontist, using pre-adjusted edgewise appliances (Mini Master Bracket, 0.022-in MBT prescription; American Orthodontics, USA). After etching the enamel surface with 37% phosphoric acid gel (Etching agent; Reliance, Itasca, Illinois, USA), a thin layer of primer (Transbond XT; 3M Unitek, Monrovia, CA, USA) was applied on the etched surfaces and adhesive resin was placed on the bracket bases. The brackets were firmly pressed in place, excessive resin was removed and light-cured for 10 seconds from incisal and gingival edges of the brackets, for a total exposure time of 20 seconds. After initial leveling and alignment, the participants were assigned to two groups, namely the fluoride group (n = 36) as controls, and the arginine group (n = 36). The participants in the fluoride group were asked to brush their teeth twice daily with a fluoride toothpaste (1450 ppm F^⁻^, Colgate^®^ Total Advanced; Colgate-Palmolive, New York, NY, USA) and those in the arginine group were requested to do the same with a toothpaste containing arginine (8% arginine and 1450 ppm F^⁻^, Colgate^®^ Sensitive Pro-Relief; Colgate-Palmolive, New York, NY, USA). Prior to the data collection phase, the participants of both groups were asked to brush their teeth with the control fluoride toothpaste. They were instructed to use a soft-bristled adult brush (Colgate^®^ Ortho; Colgate Palmolive (India) ltd.) and apply a solid strip of toothpaste across the entire length of the toothbrush bristles. The main author demonstrated brushing the front teeth in an up-and-down motion, and the back teeth in a circular motion. The participants were requested to brush their teeth accordingly for 2 minutes and refrain from chewing gums, using mouthwash, and taking antibiotics during the study.

*Data collection:* Biofilm samples were collected in two intervals, at the beginning of the study (T_0_) and 30 days later (T_1_). In order to collect overnight plaque at T_0_ and T_1_, the participants were requested to refrain from all oral hygiene in the morning and not to eat or drink 2 hours before the sampling. To remove the archwires, the elastomeric ties (American Orthodontics, USA) were carefully disengaged. Plaque specimens were collected from the labial surfaces immediately surrounding the orthodontic brackets of the right and left maxillary lateral incisors, by the same researcher using a sterilized dental scaler. Plaque samples were collected in accordance with the standardized technique described by Pellegrini et al.^20^ To prevent overloading of the instrument tip, four passes along the tooth at the bracket interface at the gingival, mesial, distal, and occlusal aspects were performed. The collected samples were individually placed into anonymously coded microtubes, which were then sealed. These were then transported to the Muhammad Rasulullah Research Tower at Shiraz University of Medical Sciences (Shiraz, Iran) for DNA isolation. To reduce the risk of bias, the laboratory personnel were unaware of the color-coding system.

### OUTCOMES (PRIMARY AND SECONDARY) AND ANY CHANGES AFTER TRIAL COMMENCEMENT

*DNA extraction:* DNA isolation was performed according to the manufacturer’s instruction, using a kit from RNA Biotechnology Company (Isfahan, Iran). Briefly, 80 µl of buffer was added to each sample, followed by thermal cycles at 75ºC for 15 min and then at 90ºC for 30 min. The samples were then put on ice for 10 min and subsequently centrifuged at 12,000 rpm for 10 min. The supernatant contained DNA. Three samples were randomly selected and amplified by polymerase chain reaction (PCR) (Taq DNA Polymerase 2x Master Mix RED, Ampliqon, Denmark) to check the primer specificity. Primer sequences of *S. mutans* (synthesized by Metabion, Munich, Germany) used in the PCR and real-time PCR are shown in Table 2. 

**Table 2:** Thermal procedure and primer sequences of *S. mutans* used in the PCR and real-time PCR.

Temperature	Time		Type of primer	Sequence
95º C	15 min.		Forward	GTGTTGATGCGGTGGATA
95º C	10 sec.	40 cycles	Reverse	GAAGGTAAGGAGTGTCGTT
60.5º C	15 sec.			
72º C	30 sec.			

*Relative quantification of S. mutans:* The quality and quantity of DNA samples were measured using the NanoDrop Spectrophotometer. Real-time PCR (SYBR green) was used to obtain the relative quantity of *S. mutans* in the samples, with the aid of the standard curve analysis. DNA amplification was determined at 99% efficiency and R^2^ of 0.99, using RealQ Plus 2x Master Mix Green (Ampliqon, Denmark) by an expert blinded to the treatment groups. To maximize the precision of the quantitation, the DNA concentration in all samples was adjusted to the same value and a certain DNA volume of all samples was taken for real-time PCR. Thermal cycles in real-time PCR are presented in Table 2.

#### INTERIM ANALYSIS AND STOPPING GUIDELINES

Not applicable.

#### STATISTICAL ANALYSIS

The data were analyzed using the Statistical Package for Social Sciences (version 15.0, SPSS Inc., Chicago, IL, USA). The homogeneity of the groups in terms of age and sex was assessed using the Chi-square test and *t*-test. Continuous variables were expressed as median and interquartile range due to the non-parametric nature of data. Mann-Whitney and Wilcoxon tests were used for statistical comparison. *P-*values smaller than 5% were considered statistically significant.

## RESULTS

### PARTICIPANTS FLOW

**Figure 1:**
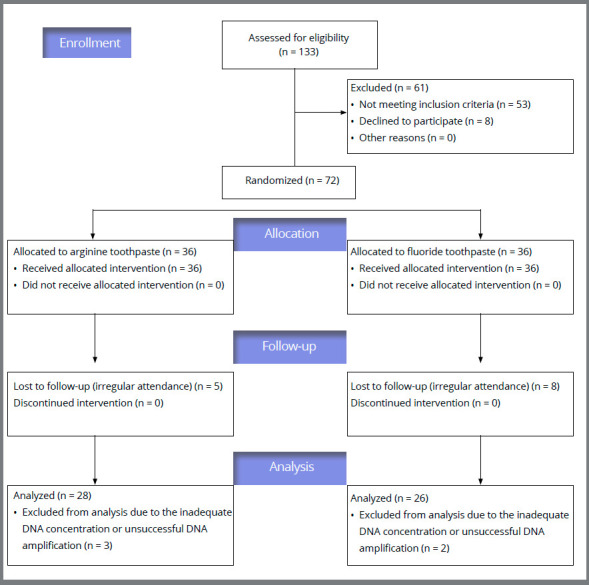
CONSORT flow diagram, displaying the progress of all participants through the trial.

During the trial, eight patients in the arginine group and ten patients in the fluoride group were excluded from the study due to irregular attendance, inadequate DNA concentration, or unsuccessful DNA amplification (Fig 1).

### BASELINE DATA

Data regarding age, sex, and duration of alignment phase of the participants are listed in Table 3. The results showed no significant difference between the study groups in terms of age and sex and duration of alignment phase. 

**Table 3:** Demographic characteristics of the samples.

Group	Age (years) mean ± SD	Sex male/female	Alignment phase (months) mean ± SD
Arginine toothpaste	22.1 ± 6.3	7/21	5.3 ± 2.1
Fluoride toothpaste	21.8 ± 5.7	6/20	4.8 ± 1.6
*p*-value	0.85^α^	0.94^β^	0.33^α^

^α^ t test results, ^β^ Chi-square test.

### OUTCOMES

**Figure 2:**
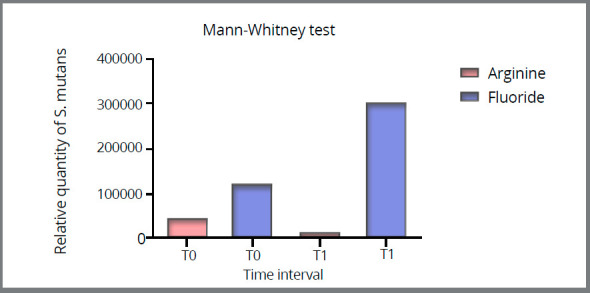
Relative number of *S. mutans* in both study groups at different time intervals ( T0: At the beginning of the study, T1: After 30 days ).

Since the variables had non-normal distribution, non-parametric tests were used in the statistical analysis. There was no significant difference between the two study groups in terms of the *S. mutans* number at T_0_ (*p*=0.8456) (Fig 2). However, the results showed that the bacterial number significantly decreased in the arginine group at T_1_ after using the toothpaste (*p*=0.0229), while there was no significant difference between the two intervals (*p*=0.1899) in the fluoride group (Table 4).

**Table 4:** Interquartile range (IQR) of the two study groups and results of Mann-Whitney and Wilcoxon tests.

Time	Group		P value (Mann-Whitney)
	Arginine IQR	Fluoride IQR	
T0	35.9-281.6	26.0-468.2	0.836
T1	22.6-152.6	15.9-264.8	0.934
P value (Wilcoxon)	0.024	0.182	

T0, at the beginning of the study; T1, after 30 days.

### ADVERSE EFFECTS

No harm or adverse effects were observed during the trial.

## DISCUSSION

### MAIN FINDINGS IN THE CONTEXT OF THE EXISTING EVIDENCE

The present clinical trial investigated the effects of toothpaste containing 8.0% arginine on *S. mutans* in dental biofilm around the fixed orthodontic brackets, and compared the results with the efficacy of regular fluoride toothpaste. It is well known that during fixed orthodontic treatment the prevalence of *S. mutans* and *lactobacilli* is increased in plaque and saliva, which may increase the risk of enamel demineralization and white spot lesions.^21^ ^-^ ^23^ In both the control and intervention group, the number of *S. mutans* was assessed at baseline and after 30 days using the real-time PCR method. Note that there was no report of any side effects or adverse effects during the trial. The results showed that the relative number of *S. mutans* significantly decreased following 30-day usage of the 8% arginine toothpaste. However, there was no difference in the number of *S. mutans* between the two sampling intervals in the case of using fluoride toothpaste. These findings were to a certain extent in line with those of previous studies.^9^ ^,^ ^15^ ^,^ ^19^ ^,^ ^24^ ^,^ ^25^ Huang et al.^17^ reported that arginine reduced the biomass of polymicrobial and *S. mutans* biofilms due to its effect on water-insoluble extracellular polymeric substances. A study by Fu et al^26^ also revealed that a desensitizing paste containing 8% arginine had significant inhibitory effects on the biofilm formation of *S. mutans* on dentine discs. On the other hand, in a double-blinded randomized clinical trial, an 8% arginine toothpaste was shown to reduce the production of lactic acid in the *in situ* plaques without changing the metabolic activity, live/dead bacteria ratio, and total biofilm biomass.^27^ Moreover, in a pilot study with nine participants using a toothpaste containing 8% arginine over 8 weeks, an increase in the arginolytic potential and a decrease in the sucrose metabolism in saliva was reported, without any significant changes in the number of *S. mutants*, neither in the saliva nor in the plaque samples.^25^ Note that these studies^25^ ^,^ ^27^ had a crossover design, small sample size, and lacked a dedicated control group. The majority of the above-mentioned studies reported that arginine maintained healthy oral biofilm and thereby reduced the risk of dental caries. However, there were some discrepancies in terms of its effect on *S. mutans* count. This could be because the inhibitory effects of arginine on *S. mutans* are not bactericidal. It has been shown that arginine interrupts the coaggregation of cells within biofilm or adhesion to substrates.^17^ A study on *S. mutans* biofilm using atomic force microscopy revealed that the production and/or composition of extracellular membrane glucans was influenced by arginine, and in turn affected their adhesion properties.^28^ This may explain the significant inhibitory effects of arginine on *S. mutans* growth in the biofilm samples collected from the tooth surfaces, which is in line with the present findings. But, none of the previous studies assessed the efficacy of 8% arginine toothpaste in patients with fixed orthodontic appliances.

As previously mentioned, the complexity of fixed orthodontic attachments and reduction in self-cleaning capacity of tooth surface during the orthodontic treatment generate more bacterial colonization and increase development of white spot lesions. Fluoride has been used as a preventive method, but it has not been enough to avoid its occurrence.^6^ So, it seems beneficial to incorporate it with the other antibacterial substances. This study in line with the previous studies demonstrating the adverse effects of arginine on the growth of *S. mutans* in patients with fixed orthodontic treatment that can have a potent influence on oral biofilm ecology and reduction in withe spot lesions. However, its exact efficacy in reduction of enamel remineralization, optimal delivery method (varnish, toothpaste, or mouthwash) and also optimal daily dose have been established neither in this study nor in the previous studies. In addition, long-term use of arginine may pose a risk of increased plaque alkalization and overgrowth of oral anaerobes such as*Porphyromonas gingivalis*, a keystone pathogen associated with periodontitis*.* ^8^ So, it remains unclear whether or not this really is beneficial for the patients.

In the present study, biofilm samples were collected from the maxillary lateral incisors since these teeth are most susceptible to enamel demineralization due to decreased salivary clearance and less space between the bracket and gingiva.^1^ ^,^ ^29^ Considering limited salivary clearance in the anterior region, plaque will mainly accumulate around the orthodontic brackets. Consequently, this region becomes a sanctuary for *S. mutans* and an ideal location for white spot lesions. Saliva samples have been used in various studies to determine the number of *S. mutans* in the oral cavity.^17^ ^,^ ^23^ ^,^ ^25^ This approach has the disadvantage that the number may not be specific to the tooth surface and could stem from multiple sources, such as previous carious lesions, the tongue, and other sites harboring the *S. mutans*. In fact, some studies have shown a difference between the number of *S. mutans* obtained from saliva and plaque samples.^30^ ^,^ ^31^

The real-time PCR method was used to evaluate the number of *S. mutans* in the biofilm samples. This was an innovative step since the method permitted the determination of even a minimal quantity of bacteria. In detecting *S. mutans*, PCR assay is more specific, compared to the conventional culture methods.^32^ A previous study confirmed the reliability and high sensitivity of PCR for bacterial quantification, even in detecting a 2-fold change in the number of microorganisms.^1^ Note that in the case of a plate count method, live and culturable microorganisms are required and the number of colonies is directly affected by the condition of the samples before plate count. These limitations do not exist in the PCR method, since the DNA of microorganisms, alive or not, can still be detected. This has an impact on the way in which the samples are stored and transported. For PCR assays, the long-term stability of frozen samples is ensured.^32^

### LIMITATIONS

Some limitations to the present study are worth noting: only patients with good oral hygiene were assessed, most participants were women, and the trial was conducted in a single center during a short period (30 days). In addition, the effect of arginine on other pathogens involved in dental caries was not evaluated. The other limitations of the study were relatively small sample size and also difficulty in controlling tooth brushing. Further clinical trials over a longer follow-up period and evaluation of the effect of prebiotics on white spot lesion formation during fixed orthodontic treatment are recommended.

### GENERALIZABILITY

The results showed that 8% arginine toothpaste decreased the number of *S. mutans* in the dental biofilm of patients undergoing fixed orthodontic treatment. However, the short duration of the trial, single-center study, and the inclusion of patients with good oral hygiene limited the generalizability of the present findings.

## CONCLUSION

Thirty days usage of toothpaste containing 8% arginine by patients undergoing fixed orthodontic treatment was more effective in reducing *S. mutans* count in dental plaque, compared to the fluoride toothpaste. 
